# Sulfur Vacancy-Rich CuS for Improved Surface-Enhanced Raman Spectroscopy and Full-Spectrum Photocatalysis

**DOI:** 10.3390/nano13010128

**Published:** 2022-12-26

**Authors:** Jiapei Hu, Yinyan Gong, Lengyuan Niu, Can Li, Xinjuan Liu

**Affiliations:** 1 College of Optical and Electronic Technology, China Jiliang University, Hangzhou 310020, China; 2 School of Materials and Chemistry, University of Shanghai for Science and Technology, Shanghai 200093, China

**Keywords:** CuS, sulfur vacancy, surface-enhanced Raman spectroscopy, photocatalysis, real-time monitoring

## Abstract

There are growing interests in the development of bifunctional semiconducting nanostructures for photocatalysis and real-time monitoring of degradation process on catalysts. Defect engineering is a low-cost approach to manipulating the properties of semiconductors. Herein, we prepared CuS nanoplates by a hydrothermal method at increasing amounts of thioacetamide (CS-1, CS-2, and CS-3) and investigated the influence of sulfur vacancy (V_s_) on surface-enhanced Raman spectroscopy (SERS) and photocatalysis performance. SERS intensity of 4-nitrobenzenethiol on CS-3 is 346 and 17 times that of CS-1 and CS-2, respectively, and enhancement factor is 1.34 × 10^4^. Moreover, SERS is successfully applied to monitor the photodegradation of methyl orange. In addition, CS-3 also exhibited higher efficiency of Cr(VI) photoreduction than CS-1 and CS-2, and removal rate is 88%, 96%, and 73% under 2 h UV, 4 h visible, and 4 h near-infrared illumination, respectively. A systematic study including electron paramagnetic resonance spectra, photoelectrochemical measurements, and nitrogen adsorption isotherms were conducted to investigate the underlying mechanism. This work may help to understand the impact of vacancy defect on SERS and photocatalysis, and provide an effective and low-cost approach for the design of multifunctional materials.

## 1. Introduction

Surface-enhanced Raman spectroscopy (SERS) is an ultra-sensitive analytical technique with promising applications in chemical and biochemical sensing including environmental pollution monitoring, food safety inspection, biomedical diagnostics, and security check [1,2,3,4]. The implementations of SERS as a preferred analysis platform demand the development of efficient and inexpensive substrate materials. It is known that the enhancement of SERS signals can be generally attributed to electromagnetic enhancement (EM), originated from enhanced local electric field due to localized surface plasmon resonance (LSPR) adsorption, and chemical enhancement (CM), derived from increased polarizability of target molecules adsorbed on substrates caused by chemical effects such as charge transfer. Typically, EM plays a more significant role in amplifying Raman signal than CM. Owning to the pronounced visible-light-responsive LSPR effect, precious metal nanostructures exhibited admirable SERS performance with enhancement factor as high as 10^7^ to 10^14^ [5,6]. However, their expensiveness and poor biocompatibility inspired seek of alternative substrate materials for SERS sensing.

Semiconductor nanomaterials are appealing candidates for the fabrication of SERS-active substrates due to their inexpensiveness, high chemical stability, as well as good selectivity [7,8]. During the past few decades, great efforts have been dedicated to investigating the SERS performance and underlying mechanism of various semiconductors such as metal nitrides [9], metal oxides [10], metal sulfides [11], MXene [12], as well as metal organic frameworks [13]. Unfortunately, the enhancement factor is usually inferior to that of precious metals, which is undesirable for sensing target molecules at very low concentrations. Defect engineering can greatly affect the adsorption of target molecules on substrates and charge transfer (CT) among them, and thus appears as an effective strategy for enhancing SERS performance [14,15,16]. Cong et al. reported that the SERS activity of tungsten oxide can be improved significantly by introducing oxygen vacancies (Vo), which was attributed to enhanced charge transfer due to coupling between oxygen defect-related states below conduction band and LUMO (HOMO) of R6G [14]. Glass et al. demonstrated that artificial creation of surface oxygen vacancies in titanium dioxide and zinc oxide result in significant enhancement of SERS signal [16]. Jiang et al. observed that defect-rich cuprous oxide hollow nanocubes with copper and oxygen vacancies exhibit improved chemisorption of dye adulterants, resulting in prominent signal enhancement of target analyte [17].

In addition to acting as SERS substrates, semiconductors have been extensively studied as photocatalysts for environmental pollution remediation, renewable energy production, and nitrogen fixation [18,19,20]. Artificial incorporation of defects in semiconductor photocatalysts is also an efficient and low-cost strategy for improving catalytic efficiency by promoting chemisorption, enhancing light absorption ability, and preventing charge recombination [21,22,23]. For instance, Zhao et al. reported that ultrathin TiO_2_ nanosheets with rich oxygen vacancies exhibited higher efficiency of nitrogen photoreduction to ammonia, and the quantum yield is 0.05% at 700 nm [24]. Singh et al. showed that oxygen vacancy-rich Cu_2_O has much higher photodegradation efficiency of MO, exhibiting a reaction rate constant 643 times that of commercial Cu_2_O [25]. Hao et al. studied a series of ZnS photocatalysts containing different amounts of zinc vacancies and found that the introduction of optimal amount of zinc vacancies can increase the visible photocatalytic hydrogen production rate from 84 to 338 μmol·g^−1^·h^−1^ [26]. Xiao’s group investigated the synergistic effect of zinc vacancies and sulfur vacancies in ZnS, and reported an optimal H_2_ generation rate of 567 μmol·g^−1^·h^−1^ under visible-light illumination due to exposed active sites, induced defect levels in the forbidden band gap, and improved separation of charge carriers [27].

Based on the above discussion, it is important to investigate the influence of vacancy defects on the improvement of SERS and photocatalytic activity, which would help to gain in-depth understanding of the correlation between microstructure and property. Moreover, the development of dual functional semiconductors, which can not only act as SERS-active substrates but only efficient photocatalysts, makes it feasible to monitor catalytic reaction on the surface of catalyst [16,28,29]. It is known that UV-visible absorption and chromatographic measurements are popular techniques to analyze photocatalytic efficiency. The general practice is to collect suspensions at given time intervals and measure the changes of remaining pollutants in solutions. Compared to conventional approaches, SERS can provide in situ information of dye degradation on catalysts, which is crucial to studying the underlying mechanism of photocatalysis.

Copper sulfide (CuS) is a typical two-dimensional transition metal chalcogenides (TMCs) consisting of alternative sheets of planar CuS_3_ triangles and CuS_4_ tetrahedrons, which are held together by interlayer van der Waals S-S bonding [30,31]. Because of its fascinating properties such as low cost, eco-friendliness, easy preparation, and excellent electronic properties, CuS has received great attention for applications in various fields including biomedical treatment, optoelectric switches, photocatalysis, and solar cells [32,33,34].Although good light-absorbing ability in visible to infrared range makes CuS an attractive candidate for photocatalytic application, its performance is severely restricted by the poor utilization rate of photo-generated carriers [34,35,36]. Moreover, to the best of our knowledge, there are few studies on the improvement of SERS signals by creation of defects in CuS substrates.

In this work, a series of CuS nanoplates were synthesized by a facile hydrothermal growth method, and the influence of sulfur vacancies (V_s_) on SERS and photocatalytic activity were studied systematically. The V_s_-rich CuS sample (CS-3) exhibited optimal SERS and photocatalytic performance. Specifically, SERS intensity of 4-nitrobenzenethiol over CS-3 substrate is 346 and 17 times that of CS-1 and CS-2, respectively, and the calculated enhancement factor is 1.34 × 10^4^. Moreover, CS-3 is utilized as full-spectrum photocatalyst for degradation of methyl orange, and simultaneously, SERS substrate for real-time monitoring the catalytic reaction process. Finally, the as-prepared CuS samples were applied to photoreduction of Cr(VI) under full-solar-spectrum irradiation, and CS-3 exhibited the optimal removal efficiency. Therefore, this work not only demonstrates the significant influence of sulfur vacancy on SERS and photocatalytic performance of CuS, but also provides an effective and low-cost approach for the design of other multifunctional metal sulfide materials.

## 2. Experimental

### 2.1. Preparation of CuS Nanoplates

4-nitrobenzenethiol (4-NBT, 95%), methyl orange (MO, 85%), copper nitrate trihydrate (CuNO, 99%), thioacetamide (TAA, ≥99%), and absolute ethanol (≥99.5%) were ordered from Aladdin (Shanghai, China) and used as received. During all the experiments, deionized water (DI, 18.2 Ω·cm) was used.

**Scheme 1:**
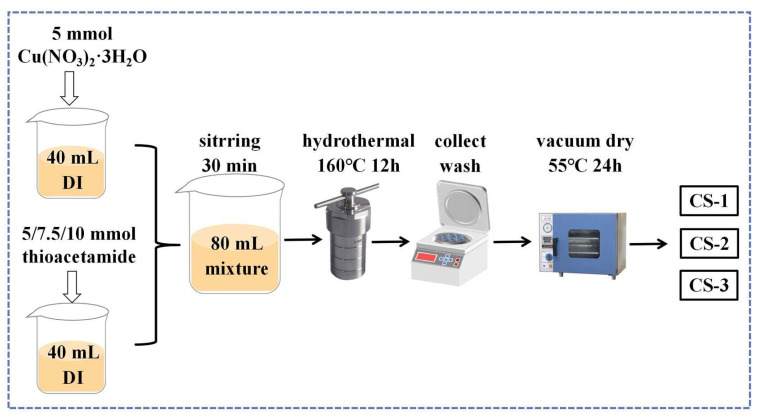
Schematic diagram of CuS synthesis procedure.

CuS nanoplates were synthesized by a simple hydrothermal growth method. Aqueous solutions of TAA (0.125, 0.188, and 0.25 M) and CuNO (0.125 M) were mixed at a volume ratio of 1:1 under stirring for 30 min. After that, 80 mL of the obtained mixtures were transferred to 100 mL Teflon-lined stainless steel autoclaves and kept at 160 °C for 12 h. After hydrothermal reaction, the synthesized precipitates were collected, washed thoroughly with ethanol and DI water, and dried in vacuum at 55 °C for 24 h. CuS nanoplates prepared at TAA solution concentrations of 0.125, 0.188, and 0.25 M were denoted as CS-1, CS-2, and CS-3, respectively. Scheme 1 illustrates the preparation procedure. The detailed description of characterization is given in Supplementary Materials.

### 2.2. SERS Performance

SERS spectra were measured on a Renishaw InVia Reflex Raman spectrometer (Gloucestershire, UK) with a 50× objective lens (NA = 0.75). The 532 nm emission line from a DPSS laser with the maximum power of 50 mW was employed as the excitation source. About 20 mg of CuS was dispersed in 20 mL of 4-NBT solutions (1 × 10^−4^ M–1 × 10^−7^ M). After being stirred for 1 h in dark, 10 μL of the suspension was drop casted on a clean Si wafer (1 × 1 cm) and dried at room temperature. The exposure time, laser power, and accumulations were set as 1 s, 1%, and 8, respectively. For the analysis, SERS spectra were collected at various locations and average results were used. Similar procedure was carried out when MO was used as the probe molecule. The exposure time, laser power, and accumulations were set as 10 s, 1%, and 8.

### 2.3. Monitoring MO Photodegradation

For the photocatalytic experiments, a 500 W mercury lamp with strong emission line at 365 nm was employed as UV light source, and the intensity (I_s_) on the sample is 101.4 mW cm^−2^.The visible light source is a 400 W halogen with strong light emission below 800 nm combined with a λ>400 nm cutoff filter, and I_s_ is 49.2 mW cm^−2^. A 300 W Xe arc lamp coupled with a λ>800 nm cutoff filter was used as NIR light source, and I_s_ equals to 41.4 mW cm^−2^. CuS (0.25 g/L) was dispersed in MO solution (2 × 10^−5^ M) by magnetic stirring in dark for 30 min to reach adsorption–desorption equilibrium. After that, the suspensions were subjected to light irradiation under ambient condition at constant stirring. At given time intervals, 10 μL of the suspension was withdrawn and dropped on Si wafer for SERS measurements. At the same time, 4 mL of the suspensions were withdrawn and filtered through a 0.22 μm membrane to remove CuS photocatalysts. The concentrations of the remaining MO in the filtrates were monitored by a 722 s UV-Vis spectrometer (Jingke, Shanghai, China).

### 2.4. Photoreduction of Cr(VI)

CuS (1 g/L) was mixed in Cr(VI) solution (50 mg/L) by magnetic stirring in dark for 30 min to reach the adsorption–desorption equilibrium. After that, the suspensions were subjected to UV, visible, and NIR light irradiation. The photoreduction rate of Cr(VI) was evaluated following the same procedure as that of MO degradation. The stability test was carried out for Cr(VI) reduction over recycled catalysts under visible light irradiation for three consecutive times and the experimental conditions were kept the same.

## 3. Results and Discussion

**Figure 1:**
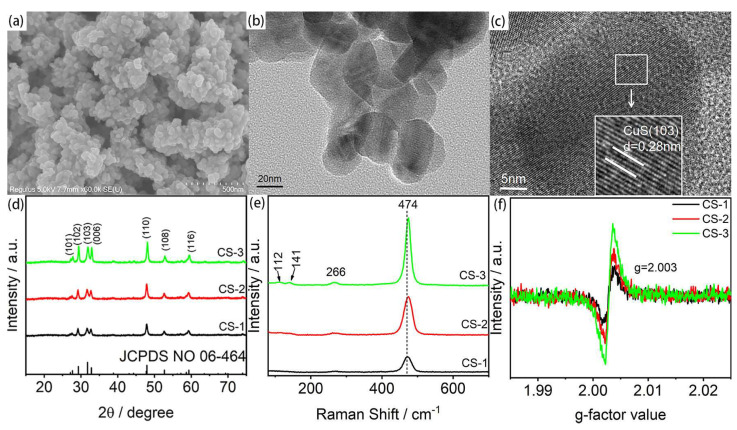
(**a**–**c**) SEM, TEM, and HRTEM images of CS-3, respectively. (**d**) XRD diffraction patterns, (**e**) Raman spectra, and (**f**) EPR spectra.

As shown in the SEM images in Figure S1a,b and Figure 1a, as-synthesized CuS samples have a cactus-like morphology assembled by nanoplates with dimension of approximately 49, 45, and 52 nm for CS-1, CS-2, and CS-3, respectively. As displayed in Figure 1b, the low-magnification TEM image of CuS-3 consistently revealed a cactus-shaped morphology consisting of nanoplates. Figure 1c presents the high-resolution TEM image of an individual nanoplate, in which the observed lattice d-spacing of 0.28 nm matched well with the (103) plane of hexagonal CuS. The crystal structures and phase purifies of CuS samples were further investigated by XRD and Raman measurements. As presented in Figure 1d, the XRD curves of CS-1, CS-2, and CS-3 can be indexed to hexagonal covellite CuS structure with a P63/mmc space group (JCPDS 06–0464). The observed Bragg reflections at 2θ = 27.57, 29.17, 31.70, 32.69, 47.89, 52.63, and 59.28°correspond to (101), (102), (103), (006), (110), (108), and (116) crystal planes, respectively. Compared to standard JCPDS pattern, the intensity of (103) reflection is reduced whereas the intensity of (110) peak is increased, suggesting preferred growth along (110) plane. Moreover, the diffraction peaks shift toward higher angle side with the increase of TAA concentration during synthesis. This shift implies lattice contraction, which is plausibly due to the formation of a higher amount of vacancies in CS-3 than CS-1.

As tabulated in Table 1, CS-3 exhibits reduced lattice constants and crystallographic unit cell volume compared to CS-1. The lack of XRD features, associated with impurity phase, and high diffraction intensity demonstrate hexagonal CuS samples with high crystallinity. Figure 1e presents the Raman spectra of as-prepared CuS samples. The observed prominent Raman peak centered at ~474 cm^−1^ originated from the S−S bond vibration (ν_SS_) of S_2_ ions occupied at the 4*e* crystallographic sites [31,37], which is highly sensitive to disorder in the sulfur sub-lattice. Compared to CS-1, the ν_SS_ vibration mode of CS-3 is positively shifted by ~2 cm^−1^. It is known that phonon confinement effect and local stress can commonly result in Raman frequency shift. According to previous studies [38,39], phonon confinement effect can cause blue shift of Raman bands by decreasing nanocrystal size. Because the size of CS-3 is no less than that of CS-1, the observed Raman peak shift is unlikely due to phonon confinement. Hence, the plausible explanation is that CS-3 contains rich vacancies, and the induced local stress cause the observed blue shift. In addition to 474 cm^−1^ peak, there are weak Raman features at ~112, ~141, and ~266 cm^−1^, which are attributed to vibration modes related to Cu-S bonding, where S ions occupy the 2*c* sites [31]. Figure 1f showed the EPR spectra of CuS nanoplates, in which the isotropic peak at g = 2.003 can be attributed to the contribution of unpaired electrons due to sulfur vacancies [40,41,42]. The stronger EPR signal in CS-3 suggests the formation of more sulfur vacancies with increasing concentration of TAA, which is beneficial for improving SERS and photocatalytic activity.

**Figure 2:**
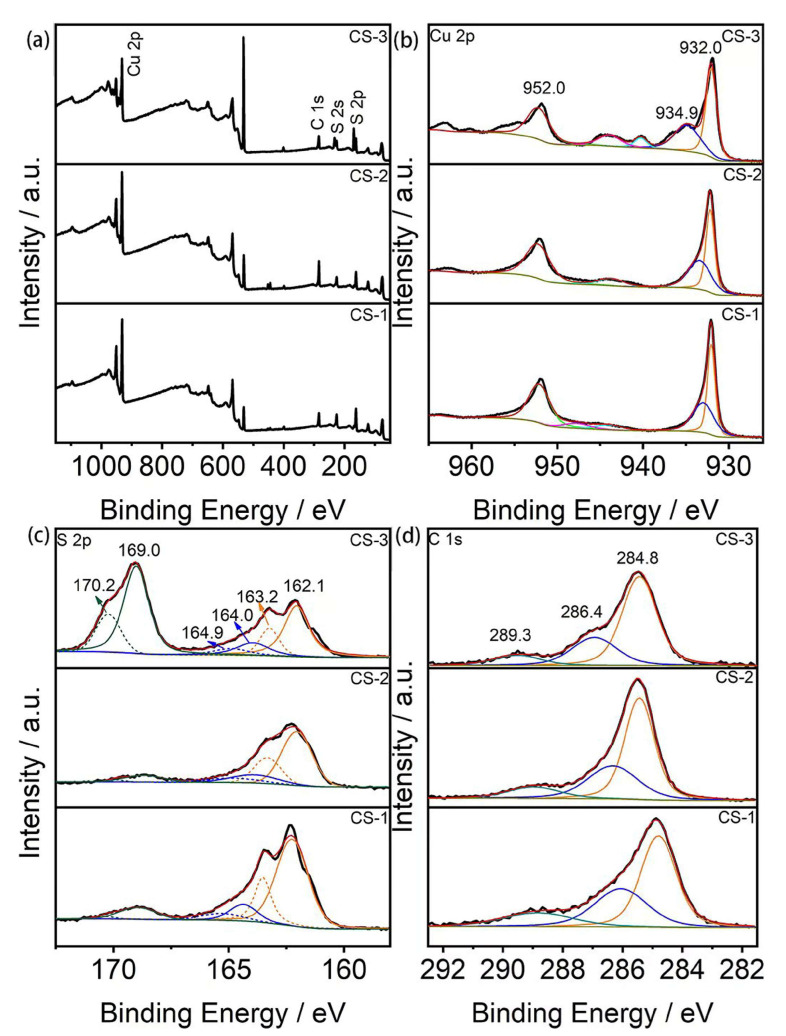
XPS spectra of CS-1, CS-2 and CS-3 (**a**) full survey, (**b**) Cu 2p, (**c**) S 2p, and (**d**) C1s.

EDX spectrum indicates that the as-made CuS nanoplates are mainly composed of copper and sulfur elements (Figure S2). Moreover, XPS measurements were performe to study the surface chemical composition and oxidation states. In the survey spectra (Figure 2a), the binding energy (BE) peaks of Cu 2p and S 2p along with C 1s and O 1s are detected, which is presumably due to the exposure of CuS nanoplates to air. Figure 2b–d showed the high-resolution Cu 2p, S 2p, and C 1s XPS spectra, respectively. The Cu 2p spectra can be deconvoluted into two BE peaks at 932.0 and 952.0 eV with a peak-to-peak energy separation of 20.0 eV, which can be ascribed respectively to Cu 2p_3/2_ and Cu 2p_1/2_ of CuS [40,41,43]. Moreover, the shoulder at 934.9 eV is presumably originated from slight oxidation of CuS, which is ascribed to CuSO_4_ [44]. The S 2p XPS spectra can be deconvoluted into peaks located at 162.1 and 163.2, corresponding to S 2p_3/2_ and S 2p_1/2_ of CuS, and peaks at 164.0 and 164.9 eV, originating from S-S 2p_3/2_ and S-S 2p_1/2_ of CuS, respectively [44,45,46]. In addition, broad BE bands at around 169.0 and 170.2 eV indicate the presence of sulfate species (e.g., SO_4_^2−^), resulted from the partly oxidization of S^2−^ during hydrothermal synthesis [44,47]. The C 1s spectra could be fitted into three peaks centered at 284.8, 286.4, and 289.3 eV, which can be attributed to C-C, C-O, and C=O, correspondingly. All the above discussed results of SEM, TEM, XRD, EPR, and XPS suggested the successful preparation of hexagonal CuS nanocrystals and CuS-3 is rich in sulfur vacancies.

**Figure 3:**
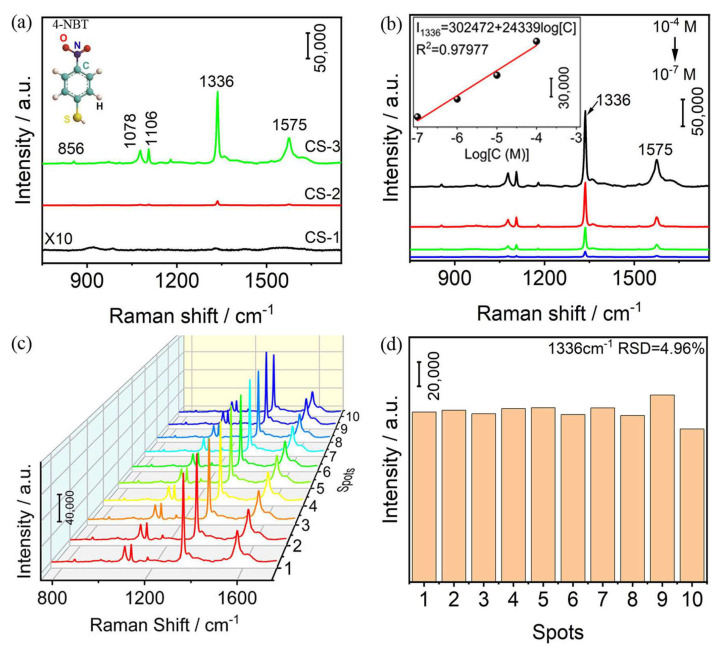
(**a**) Raman spectra of 4-NBT (1 × 10^−4^ M) on CS–1, CS–2, and CS–3; (**b**) spectra of 4–NBT at various concentrations on CS–3 from 1 × 10^−4^ M to 1 × 10^−7^ M; inset is the linear fit of 1336 cm^−1^ peak intensity with respect to Log [4–NBT concentration]; (**c**) spectra and (**d**) intensity of the 1336 cm^−1^ peak of 4-NBT on CS-3 obtained at ten randomly selected spots.

Owing to the excellent Raman scattering cross section and characteristic Raman peaks far from those of CuS substrate, which can avoid signal overlap, 4-NBT was selected as the report molecule to explore SERS activity. As displayed in Figure S3, 4-NBT powder exhibits a strong Raman peak at 1332 cm^−1^, originated from symmetric stretching vibration mode of NO_2_. In addition, there are weaker Raman bands at 856, 1100, and 1576 cm^−1^, which are assigned respectively to vibration modes of C-H wagging, C-S stretching, as well as C-C stretching of phenyl ring, [48,49]. Figure 3a showed SERS spectra of 4-NBT adsorbed on CS-1, CS-2, and CS-3 substrates at a solution concentration of 1 × 10^−4^ M. Compared to 4-NBT powder, it can be found that adsorption on CuS nanoplates results in noticeable Raman frequency shifts, i.e., the 1100 cm^−1^ peak is shifted to 1106 cm^−1^, and the 1332 cm^−1^ peak is shifted to 1336 cm^−1^, suggesting chemisorption and charge carrier transfer between target molecules and SERS substrate. Moreover, CS-3 exhibited pronounced effect on enhancing Raman signal. For the strongest Raman peak at 1336 cm^−1^, the signal intensity of CS-3 is 346 and 17 times that of CS-1 and CS-2, respectively, suggesting that creating of sulfur vacancy is beneficial to promote SERS performance of CuS nanoplates. We note that further increasing TAA content during synthesis will cause formation of secondary phase and reduced V_s_, resulting in reduced SERS signal (see Figure S4 in Supplementary Information). It can be hypothesized that rich Vs in CuS nanoplates tend to bind with sulfur atoms in –SH group of 4-NBT, enhancing adsorption of target molecules. Moreover, the presence of Vs could induce charge redistribution and increase polarizability of adsorbed molecules. In addition, sulfur vacancy can form defect levels and inhibit charge carrier recombination (see below), which makes it feasible that more photo-induced carriers participate in charge transfer process. Therefore, sulfur vacancy-rich CuS exhibits the best SERS activity.

Figure 3b show the SERS spectra of various concentrations of 4-NBT molecules adsorbed on CS-3. It is found that the intensity of 1336 cm^−1^ peak can be expressed as $I_{1336}=302,472+24,339\log\left[C\right]$ with $R^{2}=0.9797$ in the range of 1 × 10^−4^ to 1 × 10^−7^ M. The linearity of intensity versus concentration is important for sensing application. Moreover, enhancement factor (EF) is calculated to be 1.34 × 10^4^ (details of calculation are given in Supplementary Information). Furthermore, SERS spectra were recorded at ten randomly selected spots under the identical experimental conditions (Figure 3c). Similar Raman spectra were observed and the calculated RSD of the characteristic Raman peak at 1336 cm^−1^ is 4.96%, demonstrating excellent reproducibility of CS-3 substrate (Figure 3d).

**Figure 4:**
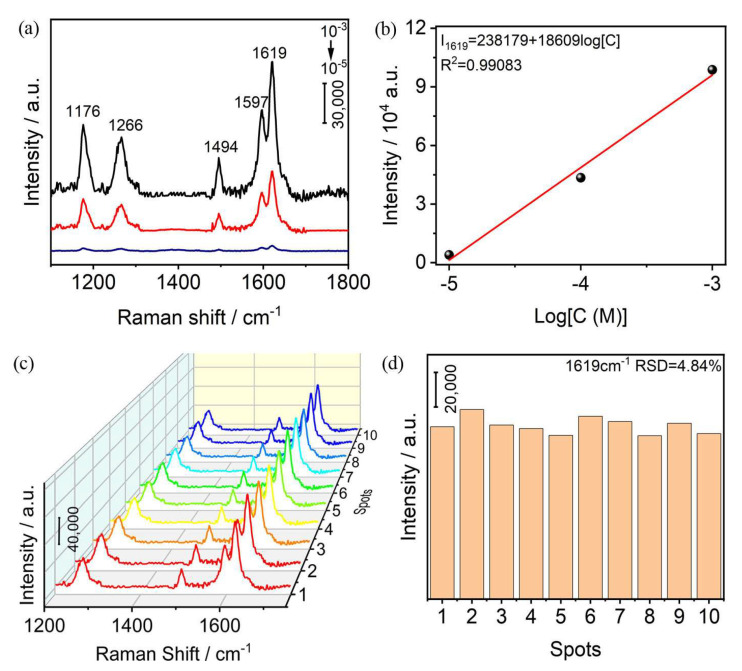
(**a**)Raman spectra of Moat various concentrations on C–3 from 1 × 10^−3^ M to 1 × 10^−5^ M; (**b**) the linear fit of 1619 cm^−1^ peak intensity versus Log[MO concentration]; (**c**) spectra and (**d**) intensity of 1619 cm^−1^ peak at of MO from different spots on CS–3.

Moreover, SERS performance of CS-3 for sensing MO, a widely used model pollutant to evaluate photocatalytic activity, was also investigated. Figure 4a presents the measured spectra of MO at various concentrations from 1 × 10^−3^ M to 1 × 10^−5^ M. There are five characteristic Raman peaks at 1619, 1597, 1494, 1266, and 1176 cm^−1^. Among them, the two strong SERS bands at 1619 cm^−1^ and 1597 cm^−1^ correspond to vibration modes of C=C stretching and bending in the benzene ring, respectively. 1494, 1266, and 1176 cm^−1^ are associated with the mixed mode of ring C-C stretching and C-H bending, C-N stretching in methyl group, and C-N=N stretching, respectively [50,51]. The intensity of 1619 cm^−1^ peak can be expressed as $I_{1619}=238,179+18,609\log\left[C\right]$ with $R^{2}=0.99083$ (Figure 4b), indicating good linearity of SERS intensity versus MO concentration. Figure 4c displays10 SERS spectra collected at randomly selected regions, which show similar characteristic bands with comparable intensity. The RSD of 1619 cm^−1^ peak is 4.84%, indicating good reproducibility of CS-3 sample (Figure 4d). The good detection linearity and reproducibility make CS-3 substrate is suitable for monitoring photocatalytic degradation of MO.

**Figure 5:**
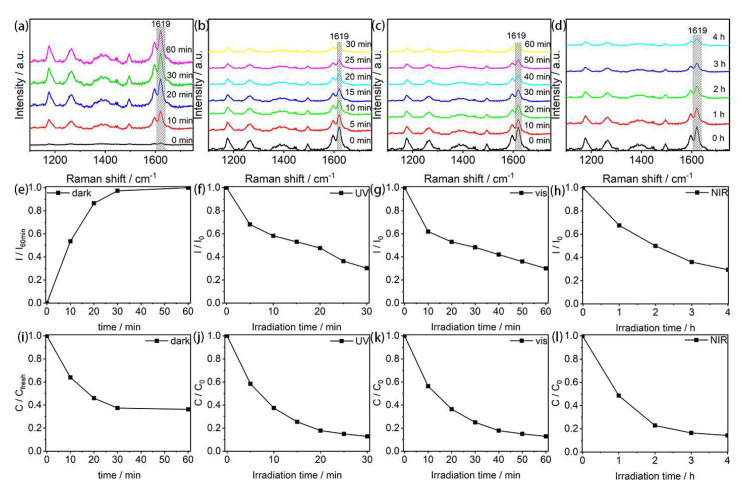
SERS spectra, intensity variation of 1619 cm^−1^ peak, and change of MO concentration in solution at various time during MO photodegradation over CS–3 under (**a**,**e**,**i**) dark environment, (**b**,**f**,**j**) UV, (**c**,**g**,**k**) visible, (**d**,**h**,**l**) NIR light illumination, respectively.

As shown in Figure S6, all the obtained samples demonstrate good light harvesting ability up to 2500 nm, and hence they are promising candidate for full-solar-spectrum responsive photocatalysis. The photodegradation of MO over CS-3 under UV, visible and NIR light irradiation has been conducted, and monitored by SERS and conventional optical absorption spectra. Figure 5a,e showed the SERS spectra and intensity variation (I/I_60 min_) of 1619 cm^−1^ band at different time intervals in dark environment. Without light irradiation, the SERS intensity of MO increases initially with prolonged time and then becomes nearly constant after 30 min. Because the Raman signal originated from adsorbed MO molecules, the observed variation of signal intensity versus time suggested that adsorption process of MO on CS-3 catalyst reached equilibrium after 30 min. Figure 5i showed the concentration changes of MO solution (C/C_fresh_) versus time monitored by a UV-Vis spectroscopy method based on Beer–Lamber law, where C is the remaining concentration of MO solution and C_fresh_ refers to the initial concentration. With increasing time, the solution concentration is first decreased due to adsorption of MO on CS-3 catalyst and then reached equilibrium after 30 min, which is in good agreement with SERS analysis. Figure 5b–d presented respectively the SERS spectra of MO at different time intervals under UV, visible, and NIR light, in which 0 min refers to the time when the adsorption process reached equilibrium and light was switched on. With increasing irradiation time, the SERS intensity is clearly decreased. Figure 5f–h showed intensity variation (I/I_0_) of 1619 cm^−1^ band versus time under UV, visible, and NIR light, correspondingly, where I is the Raman intensity of remaining MO molecules on CS-3 after illumination for a given time and I_0_ refers to Raman intensity of MO molecules on CS-3 at adsorption equilibrium. It can be found clearly that the peak intensity is decreased with increasing irradiation time, indicating photodegradation of MO molecules adsorbed on the surface of CS-3. Figure 5j–l depicted the photodegradation efficiency (C/C_0_) of MO, monitored by using a conventional UV-Vis spectroscopy method, where C is the remaining MO concentration in solution at a given time and C_0_ refer to the MO concentration at adsorption equilibrium. Consistently, the concentrations of MO solutions are decreased due to photocatalytic decomposition. The above results suggest that CuS can serve as not only full-spectrum photocatalyst but also SERS-active substrate for real-time monitoring degradation reaction. Under the same experimental conditions, we note that the decreased extent monitored by SERS measurements (I/I_0_) is different from that evaluated by UV-Vis absorption spectroscopy measurements (C/C_0_). For instance, the peak intensity is decreased to 30% of I_0_ after 30 min of UV light irradiation while the MO solution concentration is decreased to 13% of C_0_. Correspondingly, the calculated rate constants (k) are 0.036 min^−1^ and 0.069 min^−1^ (Figure S7). The difference is likely due to the fact that SERS monitoring the decreased number of MO molecules adsorbed on CS-3 while the optical absorption spectra monitoring the remained MO molecules in solution.

**Figure 6:**
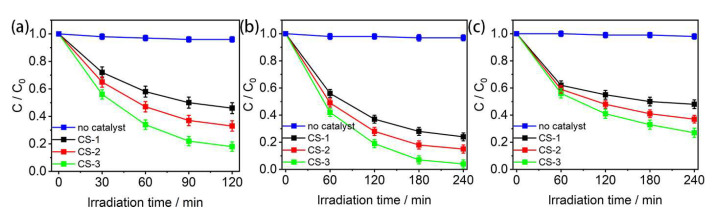
(**a**–**c**) Photoreduction of Cr(VI) over CS-1, CS-2, and CS-3 subjected to UV, visible, andNIR light irradiation, respectively.

**Figure 7:**
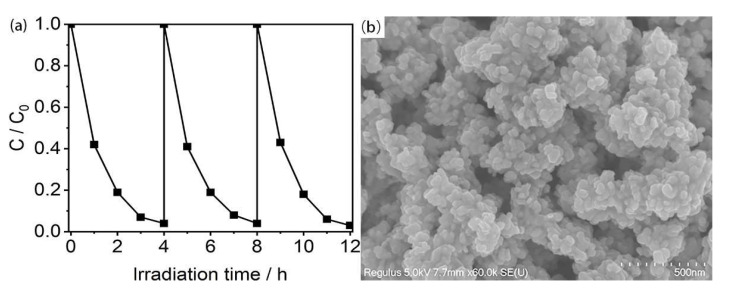
(**a**) Photoreduction of Cr(VI) over CS-3 under visible light irradiation for three consecutive runs, (**b**) SEM image of CS-3 collected after stability test.

Furthermore, photocatalytic activity of CS-1, CS-2, and CS-3 were studied by Cr(VI) reduction. Figure 6a showed time-dependent photocatalytic reduction rates of Cr(V) over CS-1, CS-2, and CS-3 under UV light irradiation. Controls showed that negligible decrease of Cr(VI) concentration in the absence of catalyst. It can be seen that the Cr(VI) reduction efficiency is increased in the sequence CS-1 < CS-2 < CS-3. In specific, the Cr(VI) reduction rate of CS-3 is 1.5 times that of CS-1 under 2 h UV light irradiation. As shown in Figure S8, the calculated k values follow the same sequence CS-1 (0.0064 min^−1^) < CS-2 (0.0093 min^−1^) < CS-3 (0.015 min^−1^). Figure 6b,c showed time-dependent photocatalytic reduction rates of Cr(V) over CS-1, CS-2, and CS-3 under visible and NIR light irradiation, respectively. As seen, CS-3 also exhibits best photocatalytic activity, and the Cr(VI) reduction rate is 1.3 and 1.4 times that of CS-1 under 4 h visible and NIR light irradiation, respectively. We note that the measured photocatalytic activity under UV is higher than visible light, which is presumably due to the different light intensity used in the experiments (101.4 mW cm^−2^ for UV and 49.2 mW cm^−2^ for visible). The reusability and stability of samples was also studied under visible light irradiation for three consecutive runs as shown in Figure 7 and Figure S9. Results indicate that CuS nanoplates are fairly stable, i.e., the Cr(VI) reduction rate remains nearly the same as that of freshly prepared sample, and no clear change of morphology after three consecutive runs is found.

**Figure 8:**
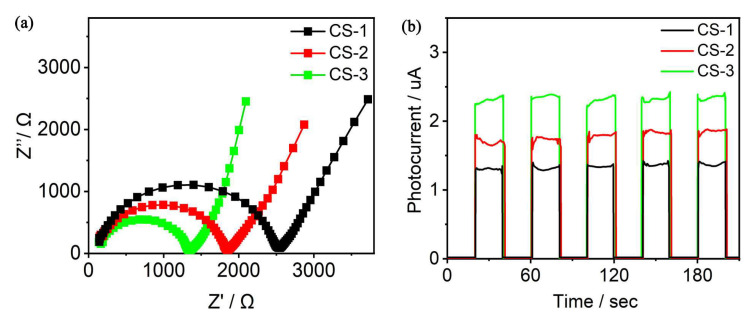
(**a**) Nyquist plots and (**b**) transient photocurrent response of CS-1, CS-2 and CS-3.

**Figure 9:**
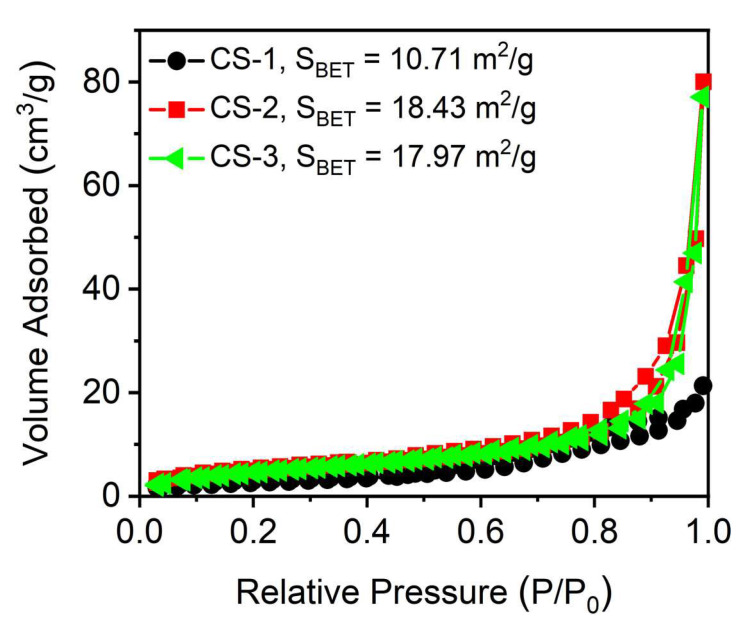
Nitrogen adsorption—desorption isotherms.

Electrochemical impendence spectra and transient photocurrent response analysis was carried out to explore the influence of sulfur vacancy on the charge carrier transfer and separation, one of the crucial factors determining photocatalytic efficiency [24,52,53,54]. As shown in the Nyquist plots in Figure 8a, CS-3 has the smallest semicircles, implying more effective suppression of photo-excited electrons and holes. Moreover, the photocurrent response follows the order CS-3 > CS-2 > CS-1, suggesting that CS-3 has better separation efficiency of charge carriers (Figure 8b). Based on the results of electrochemical measurements, the creation of sulfur vacancy defects can promote transfer and separation of charge carriers. Similar phenomena have been reported previously such as boosting photocatalytic N_2_ fixation by creation of oxygen vacancies in TiO_2_ due to promoted adsorption and charge transfer [55], and improving photocatalytic CO_2_ reduction efficiency of In_2_O_3_ via oxygen vacancies, which could increase reduction potential, suppress carrier recombination, and improve adsorption capability [54]. Since surface area and pore size could affect the accessibility of Cr(VI) to active sites and the catalytic performance, the porosity of CS-1, CS-2, and CS-3 were characterized by nitrogen adsorption—desorption measurements (Figure 9 and Table S1). It can be found that CS-3 has smaller specific surface area (17.97 m^2^ g^−1^) than CS-2 (18.43 m^2^ g^−1^), indicating that the pore structure does not play a significant role in the improved photocatalytic activity of vacancy-rich CuS.

**Figure 10:**
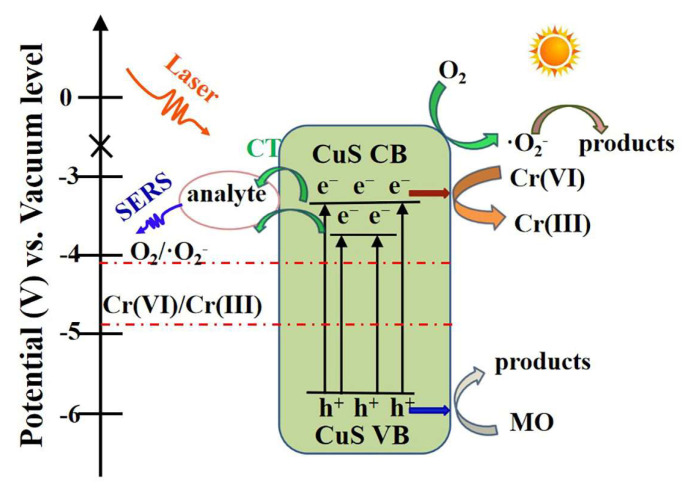
Schematic diagram illustrating enhancement mechanism of SERS and photocatalytic performance on CS–3.

Figure 10 shows schematically the feasible explanation for the enhanced photon-induced charge transfer (CT) and photocatalytic activity associated with sulfur vacancy. According to the literatures [56], the lowest unoccupied molecular orbital (LUMO) and the highest occupied molecular orbital levels of 4-NBT are −2.91 and −7.01 eV, and CuS has the conduction band (CB) and valence band (VB) at −3.67 and −5.99 eV, respectively [57]. Moreover, sulfur vacancy-related energy levels are located below CB minimum [27]. Under the 532 nm laser (2.33 eV) excitation, VB electrons in CuS are directly excited to CB, and subsequently transfer to the LUMO of 4-NBT molecules. In addition, electrons trapped at the vs. defect energy levels can also transfer to the LUMO of 4-NBT, which facilitates the CT process. The transfer of photo-excited charge carriers between target molecules and substrate would magnify Raman scattering cross-section, and consequently, result in enhancement of 4-NBT signal. Similarly, Vs defect energy levels can also promote CT between CuS and MO. The presence of sulfur vacancies can also help to promote photocatalytic activity. Surface vacancies can act as trapping sites of photo-generated charge carriers, which will inhibit charge carrier recombination. As a result, more photo-induced electrons and holes can travel to the surface of catalyst and contribute to catalytic reaction.

Therefore, sulfur vacancy plays a significant role in enhancement of not only SERS performance but also photocatalytic activity of CuS nanoplates. Sulfur vacancy-rich CS-3 exhibits best SERS sensitivity and photocatalytic activity. The dual function of CS-3 makes it suitable for applications in wastewater treatment by photocatalysis as well as monitoring by SERS analytic technique, which provide real-time information of the degradation process on the surface of catalysts, instead of the whole solution system.

## 4. Conclusions

In a summary, a series of CuS nanoplates were synthesized by a feasible hydrothermal growth method. The presence of sulfur vacancies played a crucial role in improving not only SERS performance, but also photocatalytic activity of CuS nanoplates. Sulfur vacancy-rich CuS-3 exhibits the optimal SERS intensity among all the samples and the signal intensity of 4-NBT is 346 and 17 times that of CS-1 and CS-2, respectively. Moreover, CS-3 also has the best photocatalytic performance, and the photocatalytic Cr(VI) reduction rates follow the sequence CS-3 > CS-2 > CS-1 under UV, visible, and NIR light irradiation. Furthermore, SERS sensing technique was applied to monitoring MO degradation on CuS-3, which provides an effective strategy to in situ monitor photocatalytic reaction on the surface of catalyst. This work helps to understand the impact of vacancy defect on SERS and photocatalytic activity and provides an effective and low-cost strategy for the design of multifunctional materials.

## Figures and Tables

**Table 1:** Estimated lattice parameters and unit cell volumesbased on the diffraction angles of (006) and (110) reflections.

Samples	Lattice Parameters (Å)		Cell Volume (Å^3^)
	A	C	
CS-1	3.778	9.502	135.6
CS-2	3.791	9.501	136.5
CS-3	3.791	9.483	136.2

## Data Availability

The data present in this study are available on request from the corresponding author.

## Supplementary Materials

The following are available online at https://www.mdpi.com/article/10.3390/nano13010128/s1. Figure S1SEM images of (a) CS-1 and (b) CS-2. Figure S2 EDX spectrum of CS-3. Figure S3Normal Raman spectra of (a) 4-NBT powder and (b) MO powder. Figure S4 (a) SEM image of CS-4, (b) XRD pattern of CS-4, (c) EPR spectrum of CS-4, and (d) SERS spectrum of 4-NBT (1 × 10^−4^ M) adsorbed on CS-4. In (b-d) results of CS-3 were included for comparison. Figure S5 Raman spectrum of 4-NBT solution (1 × 10^−3^ M) on Si substrate. Figure S6 UV-Vis-NIR diffuse reflectance spectra of CS-1, CS-2, and CS-3. Figure S7 Pseudo-first-order kinetic fitting curves of MO photodegradationunder UV, visible and NIR illumination monitored by SERS (a–c) and absorption spectra (d–f). Figure S8 (a, c, e) Pseudo-first-order kinetic fitting of Cr(VI) photoreductionand (b, d, f) reaction rate constants (k) under UV, visible and NIR light irradiation. Figure S9 Stability test of Cr(VI) photoreduction over (a) CS-1 and (b) CS-2.Table S1. Results of N_2_ adsorption-desorption measurements.
