# Supplementary material for: Sulfur Vacancy-Rich CuS for Improved Surface-Enhanced Raman Spectroscopy and Full-Spectrum Photocatalysis
Source: Nanomaterials (Basel). 2022 Dec 26;13(1):128. doi: 10.3390/nano13010128 (PMC9823980; doi:10.3390/nano13010128)
Supplement: Supplementary file 1 [file nanomaterials-13-00128-s001.zip › nanomaterials-2072581-supplementary.pdf]

nanomaterials

Supplementary Materials

Sulfur Vacancy-Rich CuS for Improved Surface-Enhanced Raman Spectroscopy and Full-Spectrum Photocatalysis

Jiapei Hu [1], Yinyan Gong [1,*], Lengyuan Niu [1], Can Li [1] and Xinjuan Liu [2]

1 College of Optical and Electronic Technology, China Jiliang University, Hangzhou 310020, China
2 School of Materials and Chemistry, University of Shanghai for Science and Technology, Shanghai 200093, China
* Correspondence: 13a0502075@cjlu.edu.cn

S1. Characterization of CuS nanoplates

Scanning electron microscopy (SEM) and transmission electron microscopy (TEM) images were recorded on Hitachi Regulus-8100 and FEI Tecnai G2F30, respectively. X-ray powder diffraction (XRD) measurements were performed on Bruker D2 Phaser using Cu Kα irradiation (λ = 0.154 nm). X-ray photoelectron spectra (XPS) measurements were conducted on a ThermoFisherEscalab 250Xi using Al Kα X-ray radiation (1486.6 eV), and the binding energies (BE) were referenced to the C1s line at 284.8 eV. Electron paramagnetic resonance spectra (EPR) were measured on a Bruker-A300-10/12 EPR spectrometer. UV-Vis-NIR diffuse reflectance spectra (DRS) were measured on a Perkin Elmer Lamada 750S spectrometer equipped with an integrating sphere attachment. Nitrogen adsorption isotherms were measured at 77K using a Micrometitics BELSORP-max nitrogen adsorption apparatus.

The photoelectrochemical properties of samples were tested on a CHI660E electrochemical workstation in a three-electrode configuration. To prepare working electrodes, photocatalysts and 5 wt% cellulose binder were homogeneously mixed in terpineol, coated on ITO slides (active area 1 cm^2) and vacuum dried at 60°C for 1h. Pt foil and standard calomel electrode were used as counter and reference electrodes, respectively. The electrochemical impedance spectroscopy (EIS) measurements were measured using 50 mg/L Cr(VI) solution as electrolyte. For transient photocurrent measurements, a 300 W Xe lamp with a cutoff filter (λ = 420 nm) was used as light source and 1.0 M Na_2SO_4 as electrolyte.

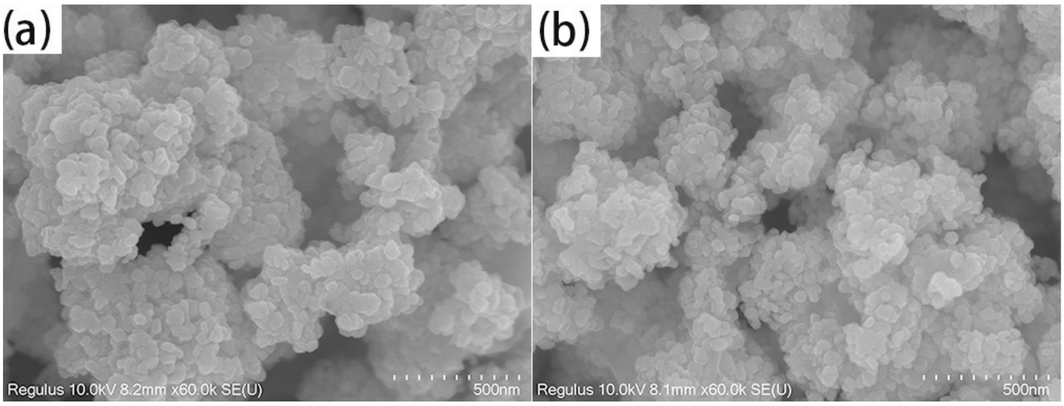

Figure S1. SEM images of (**a**) CS-1 and (**b**) CS-2.

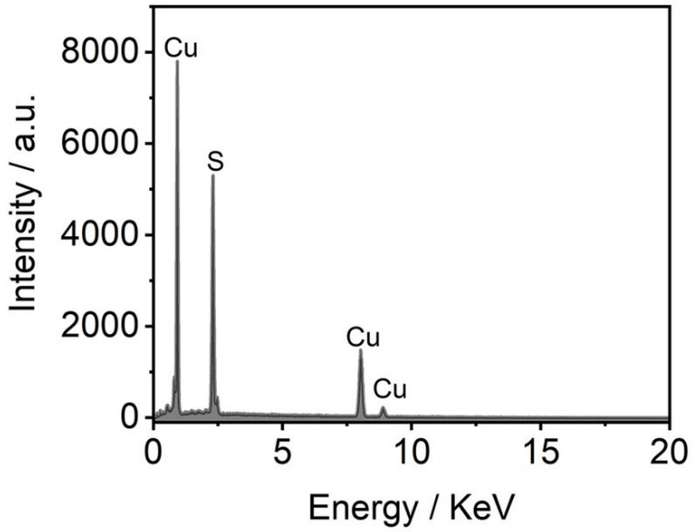

Figure S2. EDX spectrum of CS-3.

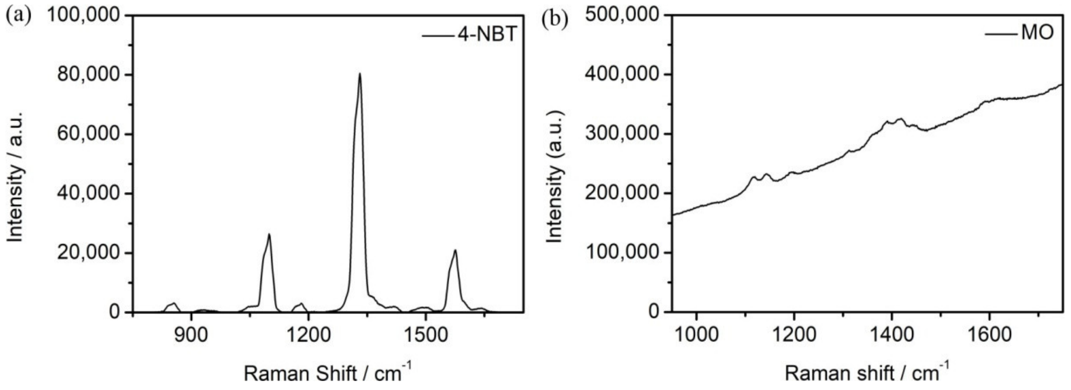

Figure S3. Normal Raman spectra of (**a**) 4-NBT powder and (**b**) MO powder.

CS-4 was prepared by further increasing the TAA content to 2.5 times that of CS-1. Figures S4a-4d showed SEM, XRD, EPR and SERS spectra of CS-4, respectively. We note that the incorporation of excess TAA during growth cause serious change of morphology and formation of secondary phase (extra diffraction peaks at 14.96, 16.59, 22.22, 28.56, 33.59, 37.01, 37.99 and 47.19°). The EPR signal intensity of CS-4 is lower than that of CS-3, implying a decreased density of S vacancy. In the SERS spectra, the ν_{NO2} mode intensity of CS-4 is reduced to 0.52 times that of CS-3. The underlying mechanism of the impurity phase formation and the reduction of S vacancy are still not understood at this stage. Since this work is mainly interested in the SERS and photocatalytic properties of CuS, we are focused on samples CS-1, CS-2 and CS-3 in the manuscript.

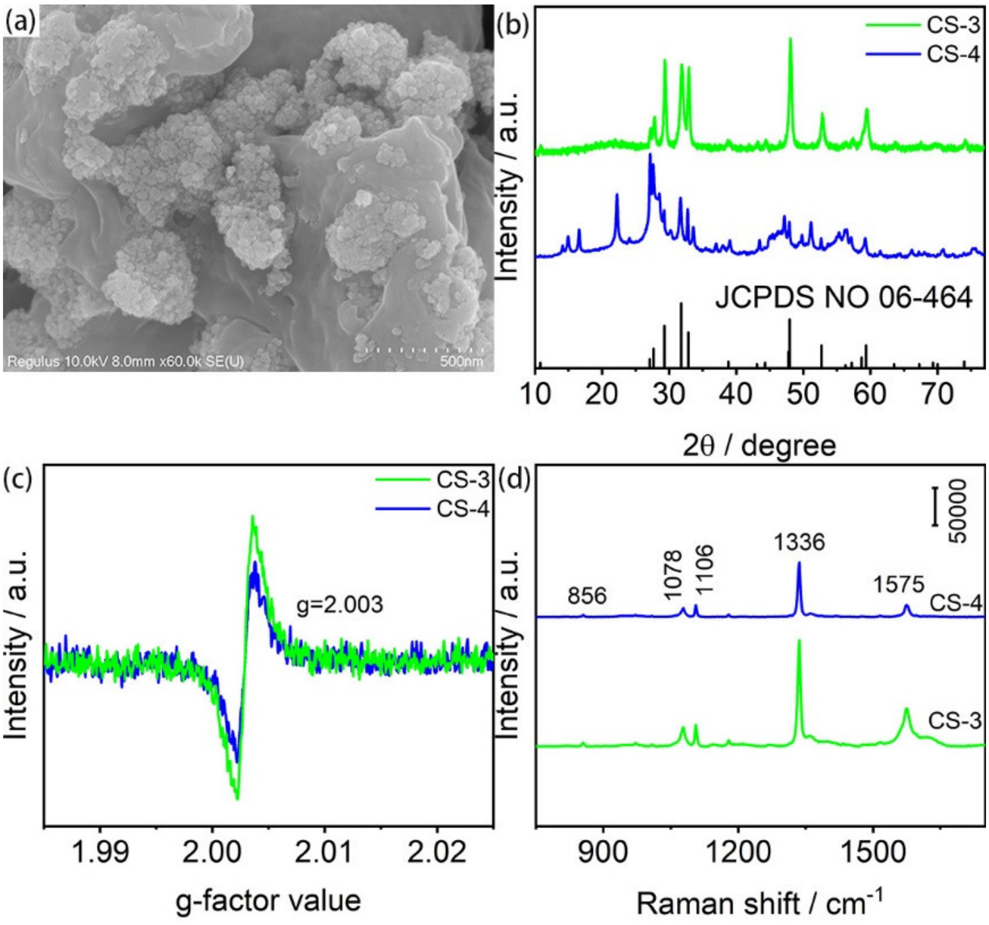

Figure S4. (**a**) SEM image of CS-4, (**b**) XRD pattern of CS-4, (**c**) EPR spectrum of CS-4 and (**d**) SERS spectrum of 4-NBT (1×10^{-4} M) adsorbed on CS-4. In (**b**–**d**) results of CS-3 were included for comparison.

S2. Enhancement factor (EF)

The SERS enhancement factor (EF) is estimated according to the following equation [1]:

$$EF = \frac{I_{SERS}}{I_{NR}} \cdot \frac{N_{NR}}{N_{SERS}} \quad (1)$$

Copyright: © 2022 by the authors. Licensee MDPI, Basel, Switzerland. This article is an open access article distributed under the terms and conditions of the Creative Commons Attribution (CC BY) license (https://creativecommons.org/licenses/by/4.0/).

where I_{SERS} and I_{NR} correspond to the integrated intensities of 4-NBT molecules adsorbed on CuS substrate and without substrate, respectively. N_{SERS} is the number of 4-NBT molecules adsorbed on CuS in the laser spot area, which can be estimated by the following equation:

$$N_{SERS} = \frac{N_{AV} \times A_{beam}}{\sigma} \quad (2)$$

where N_{AV} is Avogardro's constant, σ is the per mol area of self-assembled monolayer of molecules and equal to 3.0×10^9 cm^2/mol for 4-NBT [1], A_{beam} is the area of the focal laser, which can be calculated by the following equation:

$$A_{beam} = \pi \left(\frac{D}{2}\right)^2 = \pi \left(\frac{1.22\lambda}{2NA}\right)^2 \quad (3)$$

where D is the diameter of the diffraction-limited laser beam, $\lambda = 532$ nm is the excitation laser wavelength, and the numerical aperture of the objective lens $NA = 0.75$. Thus, the calculated laser spot area $A_{beam} = 1.87 \mu m^2$, and N_{SERS} is estimated to be 3.75×10^6. N_{NR} is the number of 4-NBT molecules in the detection area without CuS substrate. To determine I_{NR} and N_{NR}, 10 μL of 4-NBT solution ($C_{NR} = 1 \times 10^{-3}$ M) was drop cast on a clean Si wafer (1 cm × 1 cm) and dried (~6 mm). Then, N_{NR} is estimated by:

$$N_{NR} = C_{NR} \times 10\mu L \times \frac{A_{beam}}{A} \times N_{AV} = 4.1 \times 10^8 \quad (4)$$

The intensities of ν_{NO2} mode in the SERS and normal Raman spectra were used to calculate EF. $I_{SERS} = 1.67 \times 10^6$ for 4-NBT (1×10^{-4} M) adsorbed on CS-3 and $I_{NR} = 1.45 \times 10^4$ for 4-NBT (1×10^{-3} M) on Si (Figure S5). By substituting these values into Equation (1), EF is calculated to be 1.34×10^4.

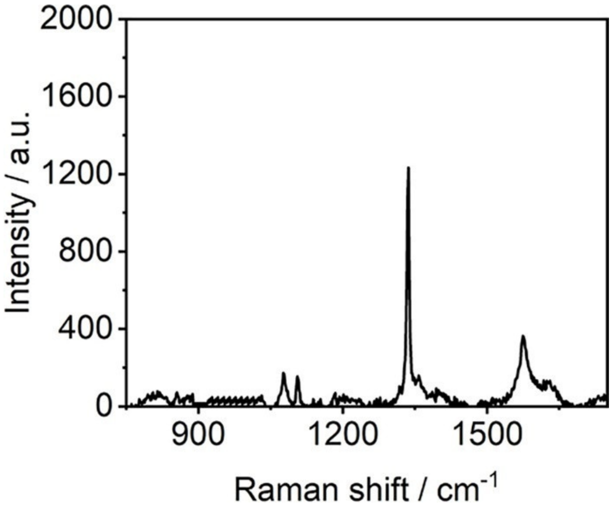

Figure S5. Raman spectrum of 4-NBT solution (1×10^{-3} M) on Si substrate.

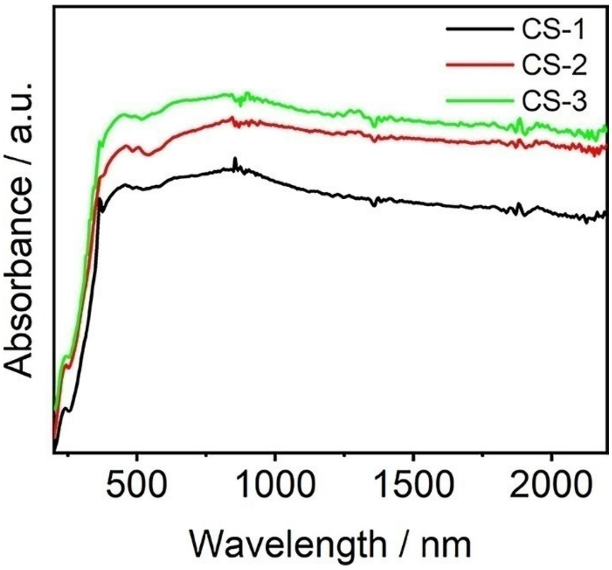

Figure S6. UV-Vis-NIR diffuse reflectance spectra of CS-1, CS-2 and CS-3.

The reaction rate constants (k) can be extracted from the linear fitting between pseudo-first-order kinetic equation and experimental results based on SERS and UV-Vis absorption spectra measurements. Figure S7a–c showed the linear fitting of $\ln(I/I_0)$ versus reaction time, and Figure S4d,f showed the linear fitting of $\ln(C/C_0)$ versus reaction time.

The calculated k values are 0.036 min^{-1} (SERS) and 0.069 min^{-1} (absorption spectra) under UV light irradiation, 0.018 min^{-1} (SERS) and 0.034 min^{-1} (absorption spectra) under visible light irradiation, as well as 0.051 min^{-1} (SERS) and 0.083 min^{-1} (absorption spectra) under NIR light irradiation.

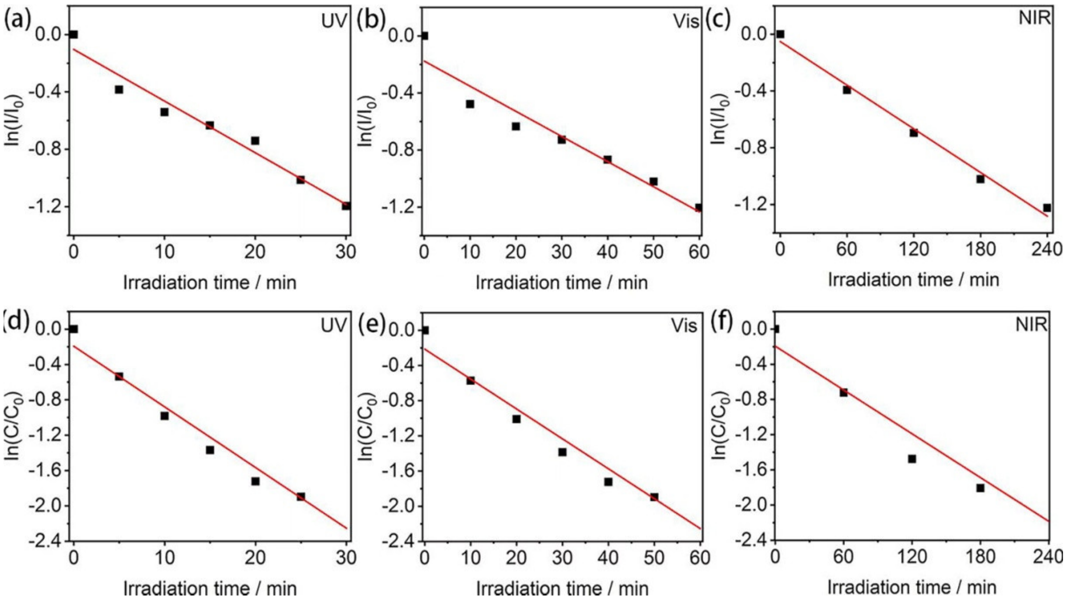

Figure S7. Pseudo-first-order kinetic fitting curves of MO photodegradation under UV, visible and NIR illumination monitored by SERS (**a**–**c**) and absorption spectra (**d**–**f**).

Figure S8a–c showed the linear fitting of ln(C/C_0) versus reaction time for Cr(VI) reduction under UV, visible, and NIR light irradiation. It can be found that the rate constants are very low in the absence of CuS photocatalysts, and equal to 0.00034, 0.00012 and 0.000084 min^{-1} under UV, visible, and NIR light irradiation, respectively. The extracted k values under UV light irradiation follows the sequence CS-1 (0.0064 min^{-1}) < CS-2 (0.0093 min^{-1}) < CS-3 (0.015 min^{-1}), demonstrating that CS-3 has the best photocatalytic activity. Under visible light irradiation, calculated k values are 0.0059, 0.0079 and 0.014 $min^{-1}$for CS-1, CS-2 and CS-3, respectively. Under NIR light, rate constants are 0.0028, 0.0039 and 0.0052 min^{-1} for CS-1, CS-2 and CS-3 respectively. Therefore, CS-3 exhibits the highest rate constants under UV, visible and NIR light irradiation.

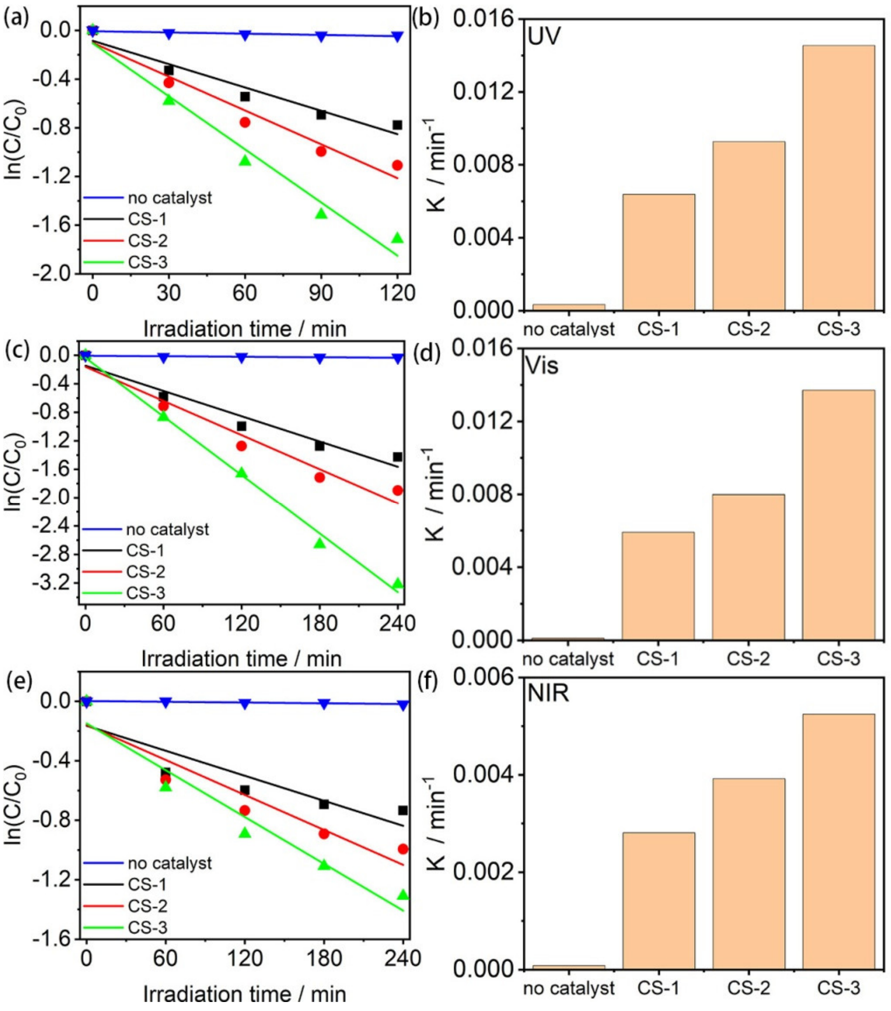

Figure S8. (**a**,**c**,**e**) Pseudo-first-order kinetic fitting of Cr(VI) photoreductionand (**b**,**d**,**f**) reaction rate constants (k) under UV, visible and NIR light irradiation.

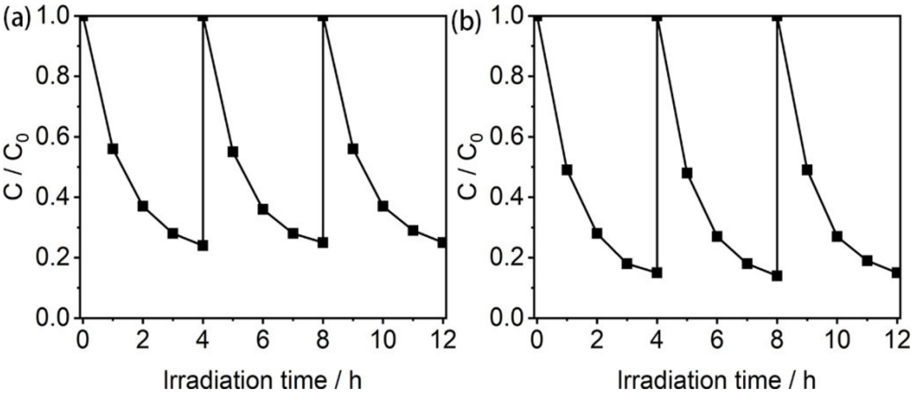

Figure S9. Stability test of Cr(VI) photoreduction over (**a**) CS-1 and (**b**) CS-2.

Table S1. Results of N_2 adsorption-desorption measurements.

Samples	Specific Surface Area (m^2/g)	Pore Volume (cm^3/g)	Pore Diameter (nm)
CS-1	10.71	0.032	12
CS-2	18.43	0.124	27
CS-3	17.97	0.120	27

References

1. Huang, J.; Zhu, Y.; Lin, M.; Wang, Q.; Zhao, L.; Yang, Y.; Yao, K. X.; Han, Y. Site-specific growth of Au–Pd alloy horns on Au nanorods: A platform for highly sensitive monitoring of catalytic reactions by surface enhancement Raman spectroscopy. *J. Am. Chem. Soc.* **2013**, *135*, 8552–8561.

Disclaimer/Publisher's Note: The statements, opinions and data contained in all publications are solely those of the individual author(s) and contributor(s) and not of MDPI and/or the editor(s). MDPI and/or the editor(s) disclaim responsibility for any injury to people or property resulting from any ideas, methods, instructions or products referred to in the content.
